# Cardiotoxin III Inhibits Proliferation and Migration of Oral Cancer Cells through MAPK and MMP Signaling

**DOI:** 10.1155/2013/650946

**Published:** 2013-04-08

**Authors:** Ching-Yu Yen, Shih-Shin Liang, Lo-Yi Han, Han-Lin Chou, Chon-Kit Chou, Shinne-Ren Lin, Chien-Chih Chiu

**Affiliations:** ^1^Department of Oral and Maxillofacial Surgery, Chi-Mei Medical Center, Tainan 710, Taiwan; ^2^School of Dentistry, Taipei Medical University, Taipei 110, Taiwan; ^3^Cancer Center, Kaohsiung Medical University Hospital, Kaohsiung Medical University, Kaohsiung 807, Taiwan; ^4^Department of Biotechnology, Kaohsiung Medical University, Kaohsiung 807, Taiwan; ^5^Department of Medicinal and Applied Chemistry, Kaohsiung Medical University, Kaohsiung 807, Taiwan

## Abstract

Cardiotoxin III (CTXIII), isolated from the snake venom of Formosan cobra *Naja naja atra*, has previously been found to induce apoptosis in many types of cancer. Early metastasis is typical for the progression of oral cancer. To modulate the cell migration behavior of oral cancer is one of the oral cancer therapies. In this study, the possible modulating effect of CTXIII on oral cancer migration is addressed. In the example of oral squamous carcinoma Ca9-22 cells, the cell viability was decreased by CTXIII treatment in a dose-responsive manner. In wound-healing assay, the cell migration of Ca9-22 cells was attenuated by CTXIII in a dose- and time-responsive manner. After CTXIII treatment, the MMP-2 and MMP-9 protein expressions were downregulated, and the phosphorylation of JNK and p38-MAPK was increased independent of ERK phosphorylation. In conclusion, CTXIII has antiproliferative and -migrating effects on oral cancer cells involving the p38-MAPK and MMP-2/-9 pathways.

## 1. Introduction

Oral squamous cell carcinoma (OSCC), the sixth most common form of cancer worldwide [1, 2], especially occurs in India, Taiwan, and Southeast Asia [3, 4]. Although many antioral cancer drugs were reported [5–9], the drug discovery against oral cancer remains a challenge.

Cardiotoxin III (CTXIII), composed of 60 basic amino acid residues, is isolated from the snake venom of Formosan cobra *Naja naja atra*. Although some anticancer drugs such as doxorubicin, anthracyclines, and trastuzumab have the well-known cardiotoxicity [10, 11], CTXIII was found to exhibit a variety of bioactivities with anticancer potential. For example, we previously found that CTXIII inhibits the cellular proliferation and induces apoptosis of various cancer cells, including breast cancer [12], leukemia cells [13], colorectal cancer [14], and oral cancer [15, 16].

The metastasis plays an important role in oral carcinogenesis [17]. However, little is known about the antimigration effect of CTXIII on oral cancer cells. In this study, we evaluated the role of CTXIII on cellular proliferation and migration of oral cancer cells Ca9-22. The role of mitogen-activated protein kinase (MAPK) family in CTXIII-induced antimigration in oral cancer cells was also investigated.

## 2. Methods

### 2.1. CTXIII Isolation

The isolation procedure of CTXIII was described previously [18]. Briefly, CTX III was purified from the venom of *Naja naja atra, *the Formosan cobrausing a chromatography on Sephadex G-50 and SP-Sephadex C-25. CTX III was dissolved in phosphate buffered saline (PBS) and filter sterilized through 0.2 *μ*m pore-size membrane filter (Millipore Corp, Bedford, MA, USA).

### 2.2. Cell Cultures

Human gingival carcinoma Ca9-22 cells [5] were cultured in DMEM-F12 medium (Gibco, Grand Island, NY). Cells were supplemented with 10% fetal bovine serum (FBS), 100 U/mL penicillin, 100 *μ*g/mL streptomycin, 0.03% glutamine, and 1 mM sodium pyruvate. Cells were kept at 37°C in a humidified atmosphere containing 5% CO_2_.

### 2.3. Growth Inhibition Test

The growth inhibition was determined by trypan blue dye exclusion assay combined with the Countess automated cell counter (Invitrogen, Carlsbad, CA, USA) as described previously [19, 20]. In brief, 1 × 10^5^ Ca9-22 cells were seeded on a 12-well plate. Cells were treated with PBS as vehicle or indicated concentrations for 24 h, respectively. After incubation, cells harvested and exposed to 0.2% Trypan blue were counted.

### 2.4. Wound-Healing Assay

Cell migration was examined by wound-healing assay as described [21]. Briefly, a total of 3 × 10^5^ Ca9-22 cells were seeded onto 12-well plates and then grown to complete confluence. A 200 *μ*L plastic pipette tip was used to scratch the culture monolayer and create a clean 1 mm wide wound area. Cells were treated with PBS (as vehicle control) or indicated concentrations of CTXIII (from 1, 3, and 5 *μ*g/mL). After incubation for 8 h, wound gaps were photographed at each time interval. The wound areas were then analyzed and calculated using the online software Wimasis (http://www.wimasis.com/; Wimasis GmbH, Munich, Germany).

### 2.5. Western Blotting

Western blot assay was described previously [22]. Briefly, cells were harvested and lysed. Lysates were centrifuged, and the protein concentration was determined. A total of 40 *μ*g protein lysates were resolved by 10% SDS-polyacrylamide gel electrophoresis (SDS-PAGE) and then electrotransferred to 0.22 *μ*m pore-size nitrocellulose membranes (Pall Life Sciences, Ann Arbor, MI). Membranes were blocked with 5% nonfat milk. Afterwards, the membranes were incubated with primary antibodies against MMP-2 (AnaSpec, no.29575), MMP-9 (AnaSpec, no.53678), phospho-JNK (Thr183/Tyr185, Upstate, no.07-175), phospho-p38 (Tyr182, Santa Cruz Biotech., sc-7973), phospho-ERK (Tyr204, Santa Cruz Biotech., sc-7976), and *β*-actin (Santa Cruz, sc-7963), their corresponding secondary antibodies, respectively. The signals were detected using a chemiluminescence detection kit ECL (Amersham Piscataway, NJ, USA).

### 2.6. Statistical Analysis

All data are presented as the means ± SD. All data were analyzed by Student's *t-*test.

## 3. Results

### 3.1. The Effect of CTXIII on Cellular Growth of Ca9-22 Cells

**Figure 1:**
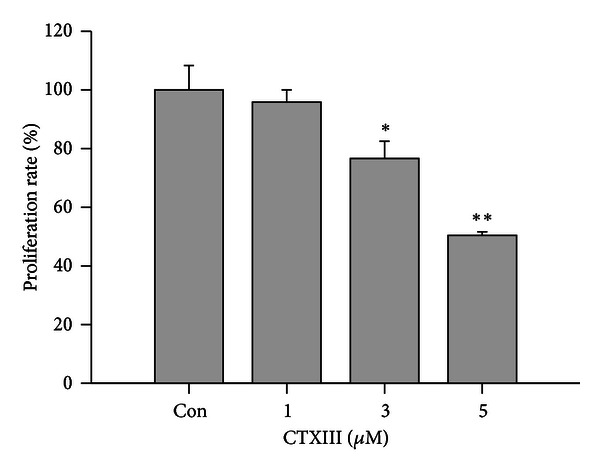
Inhibitory effect of CTXIII on proliferation of Ca9-22 cells. Cells were incubated with indicated concentrations of CTXIII (from 0, 1, 3, and 5 *μ*g/mL) for 24 h. The proliferation inhibition was determined by trypan blue exclusion assay. Data, means ± SD (*n* = 3). *P < 0.05 and **P < 0.001 for control versus CTXIII-treated, respectively.

To examine the effect of CTXIII on cell growth, Ca9-22 cells were treated with PBS as vehicle control or indicated concentrations of CTXIII (1, 3, and 5 *μ*g/mL) for 24 h, respectively. As shown in Figure 1, the cell viability was assessed by trypan blue exclusion assay, and CTXIII exerted a moderate cytotoxic effect on cell proliferation in a dose-responsive manner.

### 3.2. CTXIII Attenuates the Migration of Ca9-22 Oral Cancer Cells

**Figure 2:**
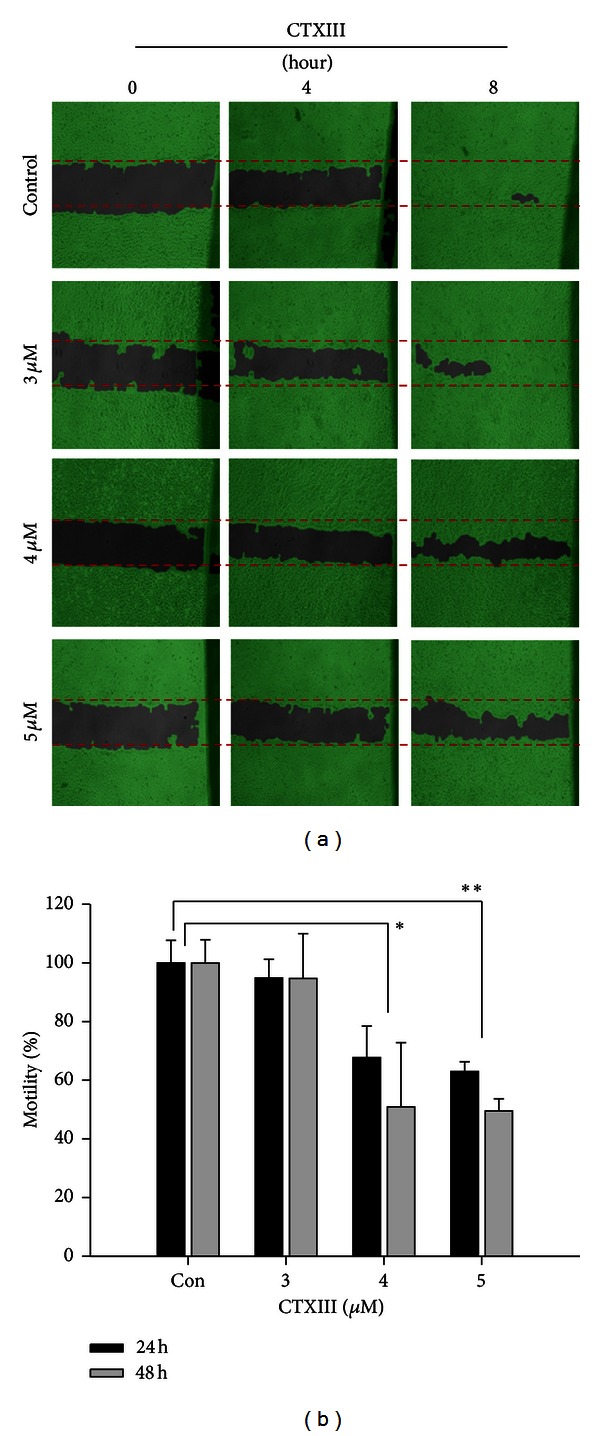
CTXIII inhibits cellular migration of Ca9-22 oral cancer cells. (a) 5 × 10^5^ cells were seeded onto a 12-well plate, and cells were scraped to create a clean 1 mm wide wound area. Cells then were treated with the indicated doses of vehicle control, 3, 4, and 5 *μ*g/mL of CTXIII for 8 hours. The wound areas were then analyzed and calculated using an online image analysis software Wimasis. (b) The quantitative results. Data, means ± SD (*n* = 3). *P < 0.05 and **P < 0.001 for control versus CTXIII treated, respectively.

Figure 2 showed that the migration of Ca9-22 oral cancer cells was significantly inhibited by CTXIII at concentrations of 2, 3, and 5 *μ*M. Additionally, the cell motility of CTXIII-treated Ca9-22 cells was inhibited in a dose-responsive and time-dependent manner.

### 3.3. Assessment of the MMP-2 and MMP-9 Expressions

**Figure 3:**
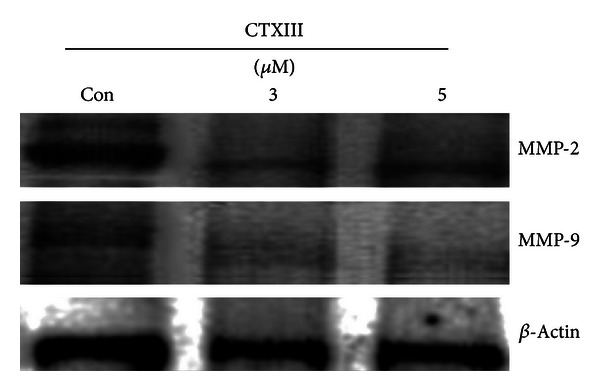
The regulation of MMP-2 and -9 expressions by CTXIII.**  **Ca9-22 cells were treated with vehicle control, 3 and 5 *μ*g/mL of CTXIII for 24 h, respectively. Two major prometastasis associated extracellular matrix metalloproteinases MMP-2 and MMP-9 were examined using the Western blot assay. *β*-Actin was used as an internal control. Each representative blot was performed in at least triplication.

To examine whether CTXIII-induced anticellular migration involves the regulation of the expression of MMPs, Ca9-22 cells treated with indicated concentrations of CTXIII (vehicle control, 3 and 5 *μ*g/mL) were subjected to the Western blotting assay. As shown in Figure 3, both MMP-2 and MMP-9 expressions were downregulated in CTXIII-treated Ca9-22 cells.

### 3.4. Assessment of the Mitogen-Activated Protein Kinase (MAPK) Signaling

**Figure 4:**
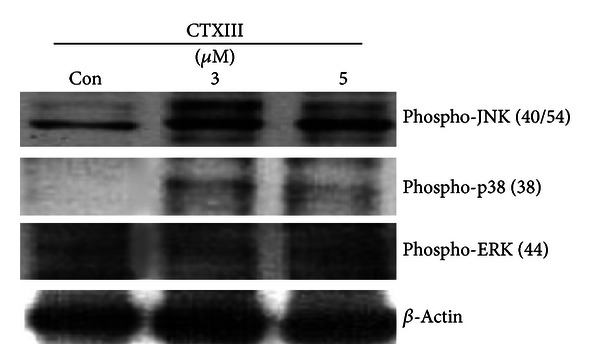
The regulation of MAPK signaling by CTXIII.**  **Ca9-22 cells were treated with vehicle control, 3 and 5 *μ*g/mL of CTXIII for 24 h, respectively. The phosphorylation levels of three major MAPK members JNK, p38, and ERK were examined using the Western blot assay. *β*-Actin was used as an internal control. Each representative blot was performed in at least triplication.

To examine whether p38-MAPK involves CTXIII-induced migration-inhibitory effect, Ca9-22 cells treated at concentrations of CTXIII (vehicle control, 3 and 5 *μ*g/mL) were subjected to the Western blotting assay. In CTXIII-treated Ca9-22 cells (Figure 4), the phosphorylation of JNK and p38-MAPK was increase, but the phosphorylation of ERK was not affected.

## 4. Discussion

In this study, the antiproliferation effect was found in CTXIII-treated oral cancer Ca9-22 cells. The inactivation of epidermal growth factor receptor (EGFR) and downstream pathways [15] and Src kinase were found to involve apoptosis and cell cycle arrest of Ca9-22 cells after CTXIII treatment [16].

For other types of cancer cells, the antiproliferation and apoptosis-inducible effects of CTXIII have been reported [12–16, 23, 24]. The detailed mechanism of CTXIII-induced apoptosis have well demonstrated, such as mitochondrial alteration, reactive oxygen species generation of neuroblastoma SK-N-SH cells [23], NF-*κ*B inactivation in breast MCF-7 cancer cells [12], and downregulation of the JAK2/PI3K signaling in breast MDA-MB-231 cancer cells [24].

In addition to the antiproliferation and apoptosis-inducible effects, we found that CTXIII can inhibit the migration of oral cancer cells. Early metastasis is a critical step for oral carcinogenesis and the overexpression of MMP-9, and extracellular matrix metalloproteinase leads to a poor prognosis of oral cancer [25]. Therefore, we found that downregulation of MMP-9 in oral cancer cells by CTXIII treatment was helpful to therapeutic effect of oral cancer. Because MMPs are highly expressed in invasive tumors, it may play a vital role in tumor invasion and metastasis [26]. For example, the levels of MMP-2 and MMP-9 proteins were related to invasion of oral cancer [27]. Several drugs such as goniothalamin [21] and 17*β*-estradiol [28] were reported to inhibit the migration of lung and colon cancer cells by attenuating MMP-2 and MMP-9 activities, respectively. Consistently, the CTXIII mediated MMP2/9 to inhibit the migration of oral cancer cells in current study.

p38-MAPK can regulate invasion by modulation of MMP-2/-9 mRNA level and zymographic activity in bladder cancer model [29]. p38-MAPK also modulated the inhibition of migration in 17*β*-estradiol-treated human colon cancer cells by inhibition of MMP-2/-9 expression [28]. Similar to the current study, CTXIII-induced inhibition of migration downregulated the p38-MAPK phosphorylation and MMP2/9 protein expression. Consistent with these findings, both MMP-2/-9 and invasive activities were enhanced by exogenous expression of wild-type MAPK-activated protein kinase 2 and inhibited by p38-MAPK inhibitor [29]. 

Although the p38-MAPK and MMP-2/-9 were mediated in CTXIII-induced inhibition of migration in oral cancer cells, the phosphorylation of ERK was not involved in current study. Similarly, TGF-*β*-induced overexpression of MMP-2 and MMP-9 was mediated by p38 MAPK but not by ERK signaling in breast cancer cells [30]. Other mechanisms also reported the antimetastatic potential of CTXIII in breast cancer, such as EGFR signaling [18] and PI3K/Akt and p38 MAPK signaling [31].

## 5. Conclusions

**Figure 5:**
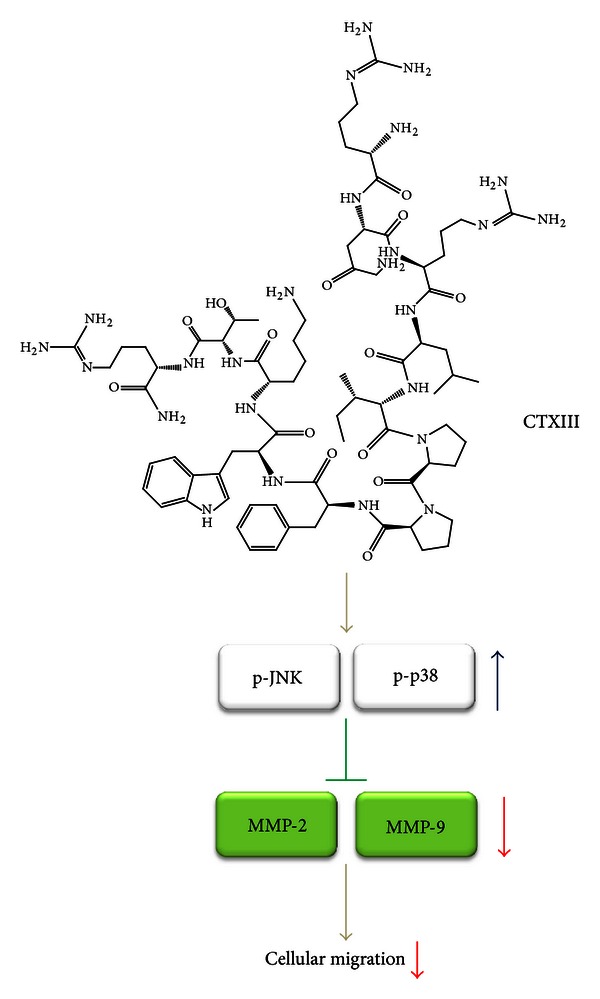
Proposed schematic mechanism of CTXIII-induced antimigration in human oral cancer cells. In current study, CTXIII exerts the antimigration potential against Ca9-22 cells in a dose-responsive manner. CTXIII causes the activation of MAPK member JNK and p38 without affecting ERK signaling. This may downregulate the expression levels of MMP-2 and MMP-9, the two major migration-associated extracellular matrix proteinases. Finally, the downregulated MMP-2 and MMP-9 expressions result in attenuating the migration potential of Ca9-22 cells.

This study demonstrates the roles of p38-MAPK and MMP-2/-9 pathways involved in the inhibition effect of proliferation and migration under CTXIII treatment in human oral cancer cells (Figure 5), and it may provide a potential oral cancer therapy.
